# Great Challenges in Catalysis and Photocatalysis

**DOI:** 10.3389/fchem.2017.00079

**Published:** 2017-10-13

**Authors:** Bunsho Ohtani

**Affiliations:** Institute for Catalysis, Hokkaido University, Sapporo, Japan

**Keywords:** catalysis, photocatalysis, Langmuir-Hinshelwood (L–H) mechanism, kinetic analysis, specialty grand challenge

Nowadays, it is difficult to find chemical products that are produced by processes without any catalytic reactions; petroleum products, plastics, textiles, pharmaceutical products, and various other chemicals are prepared through catalytic processes. If catalysts did not work for automobile exhaust, we could not live in cities due to serious air pollution. Fuel cells, which are expected to be the next-generation energy source, work efficiently only when adequate catalysts are loaded on their electrodes. Furthermore, it should be noted that almost all of the chemical reactions in living organisms are regulated by biological catalysts, i.e., enzymes. Although not to the extent of catalysis, photocatalysis has been used in various applications such as self-cleaning coatings of building materials and devices for decontamination of indoor air. Extensive studies on catalysis and photocatalysis have been carried out and will continue to be carried out with the final goal of their practical applications.

The term “activity” is used commonly in both catalysis and photocatalysis studies and the goal of those studies is to obtain higher or the highest possible activity as well as higher or 100% product selectivity. An interesting point is that the term “activity” is often recognized/described to be a property of a catalyst/photocatalyst, i.e., catalysts/photocatalysts have their own power to drive chemical reactions. For (thermal but not photo) catalyses, their principle is to reduce activation energy of a given chemical reaction, and thereby a measured rate constant, which is linked by the Arrhenius equation, can be one of the most appropriate candidates representing “activity,” and if the number or density of “active sites” can be estimated, activity can be shown by the rate constant per one active site. For photocatalyses, which are driven by photoexcitation of a photocatalyst followed by electron/positive hole transfer to reaction substrates, quantum efficiency may be “activity,” since no “active sites” can be assumed for photocatalysts. Catalysis/photocatalysis studies have therefore focused on why activity is changed or how activity is enhanced by knowing a “structure-activity correlation,” and what is predominantly discussed in scientific articles in the field of catalysis and photocatalysis is this structure-activity correlation. It is therefore necessary not only to prepare novel catalyst/photocatalyst materials and show their high activities but also to clarify what structural properties enhanced the catalytic and photocatalytic activities.

One of the possible pitfalls in scientific studies, not limited to studies on catalysis/photocatalysis, is that observed/obtained correlations are just correlations and not always causal relations. It was reported that the lifetime of Japanese women was increased almost linearly with an increase in the number of TV sets per 100 families (Kikuchi et al., 2011), but nobody would think that TV sets were the real reason for the lifetime extension (At the same time, it is also impossible, in a strict logical sense, to exclude the possibility that something emitted from TV sets extended the lifetime). Then if, for example, an almost linear relation was observed between the specific surface area (SSA) and activity of a catalyst/photocatalyst, is it possible to claim that high SSA was the reason for high activity? Unfortunately, logic does not give any suggestion for whether SSA is a number of TV sets-like something or not. Thus, even if, for example, a crystallized material exhibiting a beautiful shape in SEM images shows higher activity, an unknown/unmeasured property (properties) can be a decisive parameter for the activity. This problem might be a destiny of catalysis/photocatalysis studies, since solid materials such as powders are mainly used as catalysts/photocatalysts and the structures of those solids cannot be fully described in a strict scientific sense owing mainly to the presence of surfaces with complex and often inhomogeneous structures.

As described above, activity is generally evaluated by kinetic analysis. Many kinetic equations have been developed and used for kinetic analysis in both catalysis and photocatalysis studies, e.g., first-order rate (plots of logarithm of rate as a function of time) and surface reaction-limited reaction rate with substrates adsorbed in a Langmuir-isotherm fashion (abbreviated tentatively here as “Langmuir-adsorption rate equation;” double reciprocal plots of rate and substrate concentration). These equations are derived by assuming that the rate is proportional to the concentration of a substrate in solution (or gas phase) and on the surface, respectively, in order to estimate parameters including a rate constant. However, recently, especially in the field of photocatalysis, such kinetic equations seem to be used as only “tools” without noticing their original meaning and fundamental assumptions. For example, there have been numerous papers published in journals, even in high-level journals of high impact factors (IFs), claiming that both first-order rate and Langmuir-adsorption rate equations were successfully applied to the reported reaction without understanding the discrepancy included in it. Why were these fallacies made? One of the possible reasons is that research fields, especially those of catalysis and photocatalysis, have almost reached their maturity. In other words, negligible new concepts or assumptions generally applicable to catalytic/photocatalytic systems have recently been introduced.

In the field of photocatalysis, in which the author mainly works, the fact that the observed rate of a given photocatalytic reaction is reproduced by the above-mentioned Langmuir-adsorption rate equation is often attributed to the “Langmuir-Hinshelwood (L–H) mechanism”. However, the original meaning of the L–H mechanism proposed in the field of catalysis was completely different (Ohtani, 2010). Another, but fatal, problem appearing in papers on photocatalysis is the use of organic dyes as test substrates for decomposition. Since it is impossible to exclude the dye-sensitized mechanism in which dye molecules, not the expected photocatalyst, absorb light to be degraded by electron injection to solids (expected photocatalyst), the observed rate cannot represent solely the real photocatalytic activity of the solid. The present author has claimed this again and again over the past decade in his review papers and when acting as a reviewer of submitted papers, and he thought that this fallacy is caused by the fact that the dye-sensitized reaction satisfies so-called control experiment to check the necessity of co-presence of light, photocatalyst and substrate (Ohtani, 2016). However, he finally noticed that the authors describing the term “L–H mechanism” or using organic dyes in photocatalysis tend to rebut, when those fallacies are pointed out, by claiming that many similar papers have already been published and thereby those have been established. When has such a “majority-voting rule” been approved in science?

When the present author and his collaborators published a paper showing the inappropriate use of organic dyes as test compounds in photocatalytic reactions under visible-light irradiation (Yan et al., 2006), he did not expect its high citation since papers on photocatalysis not using dyes need not to cite that paper and papers on photocatalysis using dyes cannot cite that paper. However, the paper was actually cited many times (>200 times). When the number of citations reached 100, the present author checked the papers with citations and found, surprisingly, that more than half of the papers with citations were papers on photocatalysis using organic dyes. This could be interpreted by the assumption that the authors of such papers with mistaken citations read only the title of the paper “Is methylene blue an appropriate substrate for a photocatalytic activity test? A study with visible-light responsive titania,” as if those authors only saw a list of papers and understood the answer of this interrogative to be “yes”. Why did such a phenomenon occur?

We are now in the Internet era. This open-access journal, Frontiers in Chemistry, appeared in the Internet era and publishes papers in digital form as in other scientific journals. Thus, papers are read in digital form even if the journal is also published in printed form, and scientific papers to be checked (not “to be read”) are picked up from a list of search results, not from a table of contents of a journal issue (Therefore, an ambiguous interrogative title seems inappropriate). The above-mentioned majority-voting rule” seems like the number of “like”s in Facebook and citing a paper seems like “retweet(ing)” in Twitter.

The challenge is to achieve a breakthrough in the mature field of catalysis and photocatalysis and to correspond to the Internet-age paper-reading (checking) style. The present author, as the Specialty Chief Editor, believes that challenges can be achieved in a fundamental and orthodox way, that is, by publishing high quality papers containing not only novel insights and suggestive conclusions with little speculation, thus inspiring researchers in related fields, based on logical discussion of the results but also by not using an over-decorated/over-stressed title and abstract and providing introduction consisting of thorough and correct survey of related studies and an experimental section that enables reproduction by others.

## Author contributions

The author confirms being the sole contributor of this work and approved it for publication.

### Conflict of interest statement

The author declares that the research was conducted in the absence of any commercial or financial relationships that could be construed as a potential conflict of interest.
